# Serosurvey of anti-*Toxocara* antibodies and risk factors in adolescent and adult pregnant women of southeastern Brazil

**DOI:** 10.1371/journal.pntd.0009571

**Published:** 2021-08-04

**Authors:** Priscila de Oliveira Azevedo, Susana Zevallos Lescano, Rogério Giuffrida, Louise Bach Kmetiuk, Andrea Pires dos Santos, Sriveny Dangoudoubiyam, Alexander Welker Biondo, Vamilton Alvares Santarém

**Affiliations:** 1 Graduate College in Animal Sciences, University of Western São Paulo (UNOESTE), Presidente Prudente, SP, Brazil; 2 Institute of Tropical Medicine of São Paulo, University of São Paulo, SP, Brazil; 3 Department of Veterinary Medicine, Federal University of Parana, Curitiba, PR, Brazil; 4 Department of Comparative Pathobiology, College of Veterinary Medicine, Purdue University, West Lafayette, Indiana, United States of America; University of Iowa, UNITED STATES

## Abstract

Toxocariasis is worldwide endemic parasitic anthropozoonosis with high risk to those in in vulnerable populations and particularly during pregnancy and childhood. Although the prevalence of anti-*Toxocara* spp. antibodies has been extensively studied, risk factors of pregnant women of different ages remains to be established. This study was designed to i) assess the presence of anti-*Toxocara* spp. antibodies in pregnant women that presented to the public health system in a city of southeastern Brazil, and ii) determine the risk factors for toxocariasis in adolescent and adult pregnant women. This cross-sectional study included 280 pregnant women (71 aged up to and including 17 years [adolescents] and 209 aged 18 years and older [adults]). Pregnant women voluntarily agreed to complete a socioeconomic questionnaire and provide serum samples. Anti-*Toxocara* IgG antibodies were screened by Enzyme-Linked Immunosorbent Assay (ELISA). Univariable and multivariable logistic regression models were performed to assess the risks for toxocariasis. Overall, 20.7% of pregnant women were seropositive (33.8% of adolescents and 16.3% of adults). Prevalence in pregnant adolescents was 2.6-fold higher than in adults (Odds ration [OR]: 2.63; 95% CI: 1.42–4.86, p = 0.003). Multivariate analysis revealed that contact with soil (p = 0.01; OR = 4.76) and being in the first trimester of pregnancy (p = 0.03; OR = 0.17) had significantly greater risk of toxocariasis for adolescents, and attainment of elementary through middle school education level (p = 0.05; OR = 8.33) was a risk factor in adult pregnant women. Toxocariasis is likely underreported and neglected in adolescent pregnant women; this age group should always be monitored for toxocariasis and correspondent clinical signs, particularly at late pregnancy.

## Author summary

Toxocariasis has been described as a worldwide cosmopolitan anthropozoonosis with high risk to those in in vulnerable populations and particularly during pregnancy and childhood. Although the prevalence of anti-*Toxocara* spp. antibodies has been extensively studied, risk factors of pregnant women of different ages remains to be established. This study was designed to assess the presence of anti-*Toxocara* spp. antibodies in pregnant women presented to the public health system in a city of southeastern Brazil, and determine the risk factors for toxocariasis in adolescent and adult pregnant women. Our results show that *Toxocara* spp. seroprevalence in pregnant women may be influenced by age; with younger age identified as a risk factor for *Toxocara* spp. seropositivity. Toxocariasis is likely underreported and neglected in adolescent pregnant women; this age group should always be monitored for toxocariasis and correspondent clinical signs, particularly at late pregnancy. This data may be useful as background information for educational programs on toxocariasis prevention in pregnant women, particularly adolescents.

## Introduction

Toxocariasis has been described as a worldwide cosmopolitan and endemic parasitic anthropozoonosis, primarily transmitted to human beings by accidental ingestion of food, water or soil containing *Toxocara* spp. eggs, particularly *T*. *canis* of dogs and *T*. *cati* of cats [1,2]. Toxocariasis has been identified as one of the five neglected parasitic diseases requiring worldwide public health action [3]. Migration of *Toxocara* larvae causes various tissues of the human host in a spectrum of clinical disease [2]. Clinical toxocariasis is classified into covert, visceral, ocular, or neurotoxocariasis according to clinical signs and the organ involved [4,5] Clinical signs may vary depending on larval load, continuous reinfection, tissue distribution, and intensity of the host inflammatory response [6].

Over 1.5 billion people have been infected with soil-transmitted helminths worldwide, particularly in areas with poor sanitary conditions [7]. The prevalence of anti-*Toxocara* antibodies is higher in populations with low or lower-middle incomes and living in areas where the Human Development Index is low or medium [8].

Pregnancy in adolescence has been considered a public health problem, particularly in developing countries, with approximately 2 million girls aged under 15 years and 21 million aged between 15 and 19 years becoming pregnant worldwide every year [9]. In Brazil, adolescent pregnancy has been concentrated among females with lower education, from families with lower educational and income opportunities [10,11]. In addition to the psychosocial health problems and increased lethality for young mothers and their children [12], pregnant women living in poverty may be more likely to become infected by a variety of pathological agents, including soil-transmitted helminths, acquired by ingestion of soil and water contaminated by feces [9,13]). A recent study at a primary hospital in Ethiopia has shown that 231/448 (51.6%) of pregnant women were infected by at least one soil-transmitted helminth; women with habit of geophagia were 2.6-fold more likely infected by soil-transmitted helminths [13].

Most of the published studies on toxocariasis in pregnant women are focused on adults. Serosurveys report variable prevalence from 6.4% (18/280) worldwide [14], 7.4% (23/311) [15] in Brazil, 9.2% (91/990) in China [4], 14.5% (63/435) in nine Caribbean countries [16], 17.2% (23/134) in Greece [17], to 21.2% (40/189) in Iran [18]. Reported risk factors associated with the presence of anti-*Toxocara* spp. antibodies in pregnant women include contact with soil [17], contact with dogs and cats [14,15,18], household location [14,18], and low-income families [14].

The present study aimed to assess anti-*Toxocara* spp. antibodies in and risk factors for toxocariasis among pregnant women presented to the public health system of Presidente Prudente, southeastern Brazil.

## Materials and methods

### Ethics statement

This study was approved by the Research Ethics Committee of the University of Western São Paulo—Universidade do Oeste Paulista (Unoeste) (Protocol 4339. Plataforma Brasil: 81350117.0.0000.5515). Pregnant women who consented to participate in this study completed a questionnaire and provided a blood sample. The official consent form was signed by either the participant herself for adults or a legal guardian for underage adolescent women, as required by current Brazilian laws. All personal information obtained from subjects was kept confidential.

### Study timeline and area

The study was conducted from February 2018 through February 2019 at a public prenatal referral center located in the municipality of Presidente Prudente (22° 7’ 16.5540” S and 51° 23’ 0.2400” W), São Paulo state, southeastern Brazil. Presidente Prudente is ranked 126^th^ in number of residents (207,610), 421^st^ in per capita income (US $182.37 per month), and 25^th^ in Human Development Index (Human Development Index: 0.806) out of 5,570 Brazilian cities. At the time of this study, 29.8% of the population was earning half the minimum wage or less.

### Study design, samples, and sample collection

A cross-sectional study was conducted with 280 pregnant women enrolled during their routine prenatal visits. The sample size was calculated based on previous prevalence studies of anti-*Toxocara* IgG antibodies in pregnant women of southern Brazil [14], assuming 7.4% prevalence with 5% margin of error, 10% sample loss, and 95% confidence level.

Pregnant women voluntarily agreed to participate. Enrollment in this study involved filling out a socioeconomic questionnaire and having blood drawn for the serosurvey,

The socioeconomic questionnaire included risk factors related to age, socioeconomic status (education, family income, drinking water source, presence of sewage), gynecologic and obstetric history (gestational age, history of abortion), presence of pets, behavior (onychophagia, contact with soil), and eating habits (consumption of raw or undercooked meat). Participants were divided into two age groups: adolescents (younger than 18 years old), and adults (18 years old and above) consistent with the legal adult age of 18 years of age, according to the Brazilian Child and Adolescent Statute [19].

### Laboratory testing of samples

Blood samples were collected only once per pregnant woman by peripheral venipuncture using commercial vacuum tubes (Vacutainer, BD Co., Curitiba, Brazil). After blood collection, samples were centrifuged at 1295 g for 5 min, serum was collected and stored at -20°C until the testing.

### Antigen preparation

*In vitro* production of *T*. *canis* excretory-secretory larval antigens (TES) was based on the method described elsewhere [20], with some modifications [21]. Firstly, *T*. *canis* adult females were collected from feces of naturally infected puppies. The obtained worms were treated (5 min) with a 1% sodium hypochlorite for removing debris, followed by rinsing (3 min) in physiological solution (NaCl 0.85%). After washing, the anterior third of the worm was dissected to collect uterus and eggs. Following, eggs were kept in 2% formalin at 25°C for approximately one month for embryogenesis. After embryonating, eggs were washed in physiological solution (NaCl 0.85%) by centrifugation (559 x g for 3 min) and submitted to a rinse in 0.5% hypochlorite solution for removing the external eggs’ membrane, after washing 3 times with NaCl 0.85%, eggs were placed in an Erlenmeyer flask with glass beads and Eagle medium containing 80 mg/ml gentamicin for larval hatching. Larvae were recovered by using a modified Baermann apparatus, and then mixed with more 5 mL Eagle medium in glass tubes, followed by incubation at 37°C. Once a week, the culture supernatant, containing the TES, was collected in sterile flasks and replaced with fresh culture medium.

All the collected supernatant was treated with 200 mM of the protease inhibitor phenyl-methyl-sulfonyl fluoride (Sigma, St. Louis, MO) and stored at -20°C. About 900 ml of supernatant was concentrated 50–100 times in Amicon Ultrafiltration units (Millipore, Danvers, MA, USA), dialyzed with distilled water, centrifuged at 4°C (18,500 x g for 60 min), and filtered with 0.2 μM Millipore membranes. The protein concentration of antigen was 1.670 mg/ml, as determined by the method of Lowry et al. [22]. The resulting TES was aliquoted in vials and stored at -20°C until use. The TES prepared with *T*. *canis* likely contains both species-specific epitopes and common antigenic epitopes that are shared with *T*. *cati* (17).

### Pre-adsorption of sera with *Ascaris suum* adult worm extract

Serum samples were pre-adsorbed with *A*. *suum* adult worm extract (AWE) following an established protocol [21], to remove antibodies elicited by exposure to *Ascaris* spp. that could cross-react with *Toxocara* antigens, and, consequently, to enhance the specificity of ELISA test [23]. Adult nematodes, recovered from the intestine of slaughtered pigs, were macerated in distilled water, and NaOH was added to a final concentration of 0.15 M. After incubation at room temperature for 2 hours, the pH of the material was neutralized with 6 M HCI and centrifuged at 4°C (18,500 x g for 20 min). After removing lipids with ether, the supernatant was filtered through 0.22 μm filter membranes (Millipore Co., Burlington, MA, USA). All sera were pre-incubated at 37°C (30 min) with a final concentration of 25 μg/mL AWE in 0.01 M phosphate buffered saline (PBS, pH 7.2) containing 0.05% Tween 20 (PBS-T) (Sigma, St. Louis, MO, USA) before use in the ELISA.

### Detection of IgG anti-*Toxocara*

Serum samples were tested for IgG antibodies to TES by ELISA at a dilution of 1:200, as previously described [15]. Polystyrene 96-well microplates (Corning, Costar, New York, NY) were coated with 2.0 μg/mL of TES dissolved in 0.06 M carbonate-bicarbonate buffer, pH 9.6 (100 μL/well) for 1 h at 37°C followed by an incubation at 4°C (18 hours), and then blocked for 1 h at 37°C with 3% Molico skimmed milk PBS-Tween 5%. After sera adsorption with *A*. *suum* somatic antigen (*Ascaris* adsorbant), the adsorbant was added to wells in duplicate, incubated a 37°C, and washed three times with PBS-Tween 5%. Anti-Human IgG (Fc-specific) peroxidase antibody produced in goat (Sigma A6029) was added at a 1:5000 dilution (45 min. at 37°C), and then washed three times for 5 min.

The substrate o-phenylenediamine (0.4 mg/mL, Sigma) was used to indicate the reaction which was stopped by adding 2N sulphuric acid. Absorbance was read at 492 nm (Titertek Multiskan MCC/340, Lab-System, Finland), and a cut-off absorbance value was defined as the mean absorbance reading for 96 negative control sera plus three standard deviations. Standard positive and negative control serum and a threshold reactive serum were usedfor each tray. The antibody levels were expressed as reactivity indices (RIs) calculated as a ratio between the absorbance values of each test sample and the cut-off value, set at 0.400. A serum sample was considered positive when its RI was greater than one.

Positive sera were titrated using the same ELISA methodology as described above [15]. Sera were diluted 1:200 in the *Ascaris* adsorbant, incubated for 30 min. at 37°C and then serially diluted in the microplate with PBS-3% Molico skimmed milk -Tween 5%, incubated for 1h at 37°C, continuing with the procedure as described above. The titer of each serum was the dilution with the positive result.

### Avidity of IgG

Avidity index (AI) of IgY, was performed by a dissociation method, using a 6M urea solution as the denaturant agent [24]. The AI, expressed as a percentage, was calculated as the mean optical density (OD) of (urea-treated/urea-untreated) x 100. Values of AI up to 50 were considered low avidity (indicating recently acquired infection or recent toxocariasis) and AI exceeding 50 were considered as indicative of high avidity (past toxocariasis).

### Statistical analysis

Antibody IgG titers were transformed into Log 2 and compared with the Mann–Whitney U-test [25]. Statistical comparisons between the number of cases of recent and past toxocariasis based on AI obtained in the two groups were carried out using Fisher exact test.

## Results

### Characteristics of the study population

Of the 280 participants, 71/280 (25.4%) were adolescents (mean aged 16.3 years, range 14 to 17 years), and 209/280 (74.6%) were adults (mean aged 26.4 years, range 18 to 43 years). Participants predominantly (95.4%) had a household low income. Most of the participants (66.8%) reported having high school education. Others reporting having elementary to middle school education (25.4%) or college graduate (7.8%).

### Prevalence of anti-*Toxocara* IgG antibodies

Overall, 20.7% (58/280) **of participants were** positive for *Toxocara* spp. IgG. The clustering of different aged people showed the highest difference between seropositivity groups aged 25.5 years (S1 Fig). Seroprevalence in adolescents (33.8%) was significantly higher compared to that of adult (16.3%) pregnant women (OR: 2.63; 95% CI: 1.42–4.86, p = 0.003).

Antibody (IgG) titers raged from 1:200 to 1:800 in the adolescents and from 1:200 to 1:3200 in adult pregnant women (S1 Table). No statistical significance was observed comparing the titers of the two groups (p = 0.624).

In 19 of 21 (90.5%) adolescents and in 35 of 37 adults (94.6%) AI indicated past toxocariasis (AI > 50%). Comparison between number of recent and distant toxocariasis based on AI was not statistically significant (p = 0.615; CI 95%: 0.2394–14.146).

### Risk factor for *Toxocara* spp. infection

Univariable models. Based on univariable models, having high school and college educationwere protective factors to toxocariasis when compared to elementary/middle school education (p<0.05). This finding was observed for the group of all 280 participants (Table 1) as well as for the the adults (Table 2). Contact with soil was a risk factor associated with seropositivity in pregnant adolescents (Table 3). Moreover, late pregnancy was a protective factor for toxocariasis in adolescents (Table 3). Notably, due to the young age, only one adolescent pregnant woman had completed higher education. Although positive ELISA was not associated with previous spontaneous abortion, one out of two adolescents and three out of 17 seropositive women reported having an abortion history.

**Table 1:** Associated Risk Factors of Anti-*Toxocara* Antibodies in Pregnant Women Aged 14 to 43 Years (N = 280).

	ELISA				
Characteristic	Negative No. (%)	Positive No. (%)	OR (95% CI)	*P value*	Overall *P value*
Pregnancy stage (trimester)					0.082
First	78 (35.1)	29 (50.0)	1.0 (reference)		
Second	47 (21.2)	12 (20.7)	0.69 (0.31–1.47)	0.342	
Third	97 (43.7)	17 (29.3)	0.47 (0.24–0.92)	**0.027**	
Education level					**0.005**
Elementary/ Middle School	47 (21.2)	24 (41.4)	1.0 (reference)		
High School	155 (69.8)	32 (55.2)	0.41 (0.22–0.76)	**0.005**	
College	20 (9.01)	2 (3.45)	0.21 (0.03–0.82)	**0.022**	
Monthly family income					0.096
Up to one MW*	125 (56.3)	41 (70.7)	1.0 (reference)		
Two MW*	85 (38.3)	16 (27.6)	0.58 (0.30–1.08)	0.087	
Three MW*	12 (5.41)	1 (1.72)	0.29 (0.01–1.55)	0.171	
Water filter					1
No	167 (75.2)	44 (75.9)	1.0 (reference)		
Yes	55 (24.8)	14 (24.1)	0.97 (0.48–1.88)	0.934	
Contact with soil					0.496
No	169 (76.1)	41 (70.7)	1.0 (reference)		
Yes	53 (23.9)	17 (29.3)	1.33 (0.68–2.50)	0.399	
House dogs					0.663
No	75 (33.8)	22 (37.9)	1.0 (reference)		
Yes	147 (66.2)	36 (62.1)	0.83 (0.46–1.54)	0.555	
House cats					0.482
No	176 (79.3)	49 (84.5)	1.0 (reference)		
Yes	46 (20.7)	9 (15.5)	0.71 (0.31–1.50)	0.385	
Onychophagia					0.419
No	88 (39.6)	19 (32.8)	1.0 (reference)		
Yes	134 (60.4)	39 (67.2)	1.34 (0.73–2.52)	0.343	
Raw/undercooked meat intake					0.415
No	160 (72.1)	38 (65.5)	1.0 (reference)		
Yes	62 (27.9)	20 (34.5)	1.36 (0.72–2.51)	0.334	

*MW: minimum wage

**Table 2:** Associated Risk Factors for Anti-*Toxocara* Antibodies in Adult Pregnant Women Aged 18 Years and Older (N = 209).

Characteristic	Bivariate analysis				Multivariable analysis	
	ELISA		OR (95% CI)	*P value*	OR (95% CI)	Overall *P value*
	Negative No. (%)	Positive No. (%)				
Pregnancy stage (trimester)						
First	60 (28.7)	13 (6.2)	1.0 (reference)		1.0 (reference)	
Second	34 (16.3)	7 (3.3)	1.04 (0.39–3.06)	0.92	0.36 (0.08–1.35)	0.14
Third	81 (38.8)	14 (6.7)	1.34 (0.58–3.17)	0.48	0.17 (0.03–0.72)	**0.03**
Education						
Elementary/Middle School	28 (13.4)	12 (5.7)	1.0 (reference)			
High School	128 (61.2)	21 (10.0)	0.38 (0.17–0.89)	0.027		
College	19 (9.1)	1 (0.5)	0.14 (0.01–0.82)	0.026		
Monthly family income						
Up to one MW*	93 (44.5)	21 (10.0)	1.0 (reference)			
Two MW*	73 (34.9)	12 (5.7)	0.73 (0.33–1.57)	0.43		
Three MW*	9 (4.3)	1 (0.5)	0.55 (0.02–3.26)	0.57		
Water filter						
No	132 (63.2)	24 (11.5)	1.0 (reference)			
Yes	43 (20.6)	10 (4.8)	1.29 (0.54–2.86)	0.55		
Contact with soil						
No	133 (63.6)	29 (13.9)	1.0 (reference)		1.0 (reference)	
Yes	42 (20.1)	5 (2.4)	0.56 (0.18–1.44)	0.24	4.76 (1.47–17.33)	**0.01**
House dogs						
No	65 (31.1)	13 (6.2)	1.0 (reference)			
Yes	110 (52.6)	21 (10.0)	0.95 (0.45–2.08)	0.89		
House cats						
No	143 (68.4)	28 (13.4)	1.0 (reference)		1.0 (reference)	
Yes	32 (15.3)	6 (2.9)	0.97 (0.34–2.43)	0.96	0.25 (0.05–1.00)	0.07
Onychophagia						
No	79 (37.8)	12 (5.7)	1.0 (reference)			
Yes	96 (45.9)	22 (10.5)	1.5 (0.70–3.33)	0.29		
Raw/undercooked meat intake						
No	130 (62.2)	20 (9.6)	1.0 (reference)			
Yes	45 (21.5)	14 (6.7)	2.02 (0.92–4.33)	0.078		

*MW: minimum wage

**Table 3:** Associated Risk Factors for Anti-*Toxocara* Antibodies in Pregnant Adolescents Aged 14 to 17 Years (N = 71).

Characteristic	Bivariate analysis				Multivariable analysis	
	ELISA		OR (95% CI)	*P value*	OR (95% CI)	Overall *P value*
	Negative No. (%)	Positive No. (%)				
Gestational Age (trimester)						
First	18 (25.4)	16 (22.5)	1.0 (reference)		1.0 (reference)	
Second	13 (18.3)	5 (7.0)	0.45 (0.12–1.49)	0.195	0.88 (0.30–2.43)	0.81
Third	16 (22.5)	3 (4.2)	0.22 (0.04–0.84)	0.025	0.68 (0.29–1.61)	0.38
Education						
Elementary/Middle School	20 (28.2)	12 (16.9)	1.0 (reference)		1.0 (reference)	
High School	27 (38.0)	11 (15.5)	1.46 (0.53–4.08)	0.45	0.39 (0.17–0.92)	**0.03**
College	0 (0.0)	1 (1.4)	NC	NC	0.12 (0.01–0.69)	0.05
Monthly family income						
Up to one MW*	32 (45.1)	20 (28.2)	1.0 (reference)			
Two MW*	12 (16.9)	4 (5.6)	1.82 (0.53–7.50)	0.32		
Three MW*	3 (4.2)	0 (0.0)	NC	NC		
Water filter						
No	35 (49.3)	20 (28.2)	1.0 (reference)			
Yes	12 (16.9)	4 (5.6)	0.60 (0.15–2.02)	0.422		
Contact with soil						
No	36 (50.7)	12 (16.9)	1.0 (reference)			
Yes	11 (15.5)	12 (16.9)	3.20 (1.12–9.46)	0.03		
House dogs						
No	10 (14.1)	9 (12.7)	1.0 (reference)			
Yes	37 (52.1)	15 (21.1)	0.46 (0.15–1.38)	0.162		
House cats						
No	33 (46.5)	21 (29.6)	1.0 (reference)			
Yes	14 (19.7)	3 (4.2)	0.35 (0.07–1.26)	0.114		
Onychophagia						
No	9 (12.7)	7 (9.9)	1.0 (reference)			
Yes	38 (53.5)	17 (23.9)	0.58 (0.18–1.9)	0.36		
Raw/undercooked meat intake						
No	30 (42.3)	18 (25.4)	1.0 (reference)		1.0 (reference)	
Yes	17 (23.9)	6 (8.5)	0.60 (0.18–1.76)	0.36	2.04 (0.92–4.48)	0.08

*MW: minimum wage

Multivariable analysis. Based on the results of the univariate analysis, multivariate logistic regression analysis for the adolescent women included gestational age, contact with soil, and contact with dogs and cats. For adult women, pregnancy stage, level of education, and raw or undercooked meat intake were selected for further analysis. The multivariate analysis retained the same predictors observed in univariable models for both groups, adolescents and adults.

ROC curves showing the accuracy of the model are presented in S1 Fig.

## Discussion

This study showed a higher *Toxocara* spp. exposure in adolescents compared to adult women during pregnancy, with adolescents approximately 2.6-fold more likely to be seropositive for *Toxocara* spp. Although a similar study has compared cases in the Brazilian public health system stratified by age groups during pregnancy, no statistical differences were found at the time, even when ages were regrouped in adolescence and adulthood pregnancy [14]. The overall prevalence in a prior study f14] was 18/280 (6.4%) but was 58/280 (20.7%) in our study. The prior study may have been unable to find significant differences in associated risk factors due to the low number of positive cases.

The divergent outcome between these two Brazilian studies may be due to different population characteristics of Presidente Prudente, a southeastern countryside city, compared with Rio Grande, a southern shoreside city. Surprisingly, Presidente Prudente and Rio Grande a similar size population (227,072 and 211,005, respectively), Gross Internal Product (US $6,166.41 and US $7,831.79, respectively [exchange rate: US $1.00 to R $5.62]), and Human Development Index (0.806 and 0.878, respectively). Only elevation (475 versus 6 meters above sea level), city area (2,709.53 versus 562.8 inhabitants per square kilometer), and population density (73 versus 400 per square kilometer) were clearly different [26].

A recent worldwide meta-analysis, showed a 62,927/265,327 (19.0%) pooled seroprevalence, also concluded that being younger is a risk factor for toxocariasis [8]. Despite the similarity to to the 58/280 (20.7%) overall seroprevalence, the meta-analysis population was 24/71 (33.8%) were adolescents and 34/209 (16.3%) adults. Since reported *Toxocara* spp. seroprevalence in pregnant women, using ELISA test, ranges worldwide from 18/280 (6.4%) [15] to 40/189 (21.2%) in Iran [18], our results herein of 24/71 (33.8%) seropositive in adolescent women is the highest *Toxocara* spp. prevalence reported to date. Similarly, the prevalence in Brazilian children attending health facilities of 28/252 (11.1%) [27] to 386/1199 (32.2%) has also shown an association of young age to high toxocariasis seroprevalence [28].

ELISA assays have been widely adopted as the assay of choice for disease detection, mapping seroprevalence, and epidemiological studies, although ELISA results alone does not allow for differentiation between an recent and past toxocariasis due to the IgG persistence [29]. Thus, we evaluated the IgG avidity in seropositive pregnant women to distinguish the recent from past toxocariasis [24]. In our study, we have no found difference between IgG antibody levels in adolescent and adult pregnant women. The avidity in the majority of seropositive pregnant evaluated in our study was high, corroborating other studies [24,30,31]. In Brazil, a study involving children (n = 1309) living in poor areas, showed high avidity of IgG anti-*Toxocara* antibodies in 98.2% of the 633 seropositive children [32]. Our results suggest that independent of the age of an adolescent, the pregnant women probably have persistent antibodies indicating a past infection.

Contact with soil was found to be a risk factor for toxocariasis among pregnant adolescents (p = 0.046), but not for pregnant adults (p = 0.34), corroborating a previous meta-analysis of *Toxocara* spp. seroprevalence [8], Soil contact is a risk factor of *Toxocara* spp. exposure in children and non-pregnant adolescents [33–36], and in adults [37–39]. In addition, a previous study showed soil contamination with *Toxocara* spp. in different public parks throughout the year in President Prudente, suggesting favorable climatic conditions for eggs [40]. Although onychophagia and eating soil were not statistically significant risk factors in this study, poor hand hygiene does provide higher exposure to contaminated soil in children [39], and may lead to re-infection with limited treatment efficacy [41,42].

Despite mostly participants in this study having a pet at home, no association with *Toxocara* spp. seropositivity was observed. Contact with dogs has been previously identified as a risk factor for toxocariasis [4,8,16,27,35], including in pregnant women [14,15]. A systematic review and meta-analysis of global *Toxocara* eggs in public places identified 21% (13,895/42,797) of studied areas such as parks, playgrounds, and schools to be potentially contaminated with *Toxocara* spp. eggs [43]. Thus, our finding a lack of association between having pets at home and toxocariasis may indicate outdoor contact with contaminated soil.

This study showed lower antibody titers in the final trimester of pregnancy only in adolescents. Hemodynamic and immunological changes have been associated with decreased anti-*Toxocara* IgG detection by ELISA in late pregnancy [14,15]. As this finding was not observed in this study or in adult pregnant women of China [4], further studies should be conducted to establish whether anti-*Toxocara* spp. titers decrease at late pregnancy.

Although ingestion of raw or undercooked meat has been identified as a risk factor for toxocariasis [4,8,44] and approximately one-third of the participants reported eating raw or undercooked meat, we found no association with seropositivity. This is most likely due to Presidente Prudente’s high Gross Internal Product and Human Development Index, where commercial meat is generally handled under rigorous state or federal sanitary inspections.

The presence of anti-*Toxocara* spp. antibodies was not found to be associated with a history of spontaneous abortion, corroborating other surveys [14,15]. Although soil-transmitted helminths may compromise fertility and gestation in human beings [45], only naturally transplacental transmission of toxocariasis has been reported in humans [46], with transplacental and/or transmammary toxocariasis transmission observed in animal models under experimental conditions [47,48].

## Conclusion

In conclusion, results herein have demonstrated that *Toxocara* spp. seroprevalence in pregnant women may be influenced by age; with younger age an associated risk factor for exposure to and infection with *Toxocara* spp. The absence of a difference in IgG antibodies levels of adolescent and adult pregnant women suggests that independent of the age of adolescents, the pregnant women probably have persistent antibodies indicating a past infection. In addition, independent analysis of adolescent and adult pregnant women may provide distinct risk and protective factors. This data may be useful as background information for educational programs on toxocariasis prevention in pregnant women, particularly adolescents.

## Supporting information

S1 Fig Receiver operating characteristic (ROC) curve assessing the accuracy of the multivariate logistic regression model for predicting seropositivity for anti-*Toxocara* spp. antibodies in pregnant adolescents South-East (top; area under curve (AUC): 0.7611; 95% CI: 0.6409–0.8813) and adults (bottom; AUC: 0.6735; 95% CI: 0.5697–0.7774) attending Public Health System. South-East, Brazil. 2020.
(DOCX)

S1 Table Results of anti-*Toxocara* IgG antibodies titers and avidity index (AI) of IgY in 280 pregnant women (14 to 43 years old). (DOCX)
