# Supplementary material for: Serosurvey of anti-Toxocara antibodies and risk factors in adolescent and adult pregnant women of southeastern Brazil
Source: PLoS Negl Trop Dis. 2021 Aug 4;15(8):e0009571. doi: 10.1371/journal.pntd.0009571 (PMC8336820; doi:10.1371/journal.pntd.0009571)
Supplement: S1 Fig — South-East, Brazil. 2020. (DOCX) [file pntd.0009571.s001.docx]

**Fig. S1.** Receiver operating characteristic (ROC) curve assessing the accuracy of the multivariate logistic regression model for predicting seropositivity for anti-*Toxocara* spp. antibodies in pregnant adolescents South-East (top; area under curve (AUC): 0.7611; 95% CI: 0.6409-0.8813) and adults (bottom; AUC: 0.6735; 95% CI: 0.5697-0.7774) attending Public Health System. South-East, Brazil. 2020.
