# Supplementary material for: Serosurvey of anti-Toxocara antibodies and risk factors in adolescent and adult pregnant women of southeastern Brazil
Source: PLoS Negl Trop Dis. 2021 Aug 4;15(8):e0009571. doi: 10.1371/journal.pntd.0009571 (PMC8336820; doi:10.1371/journal.pntd.0009571)
Supplement: S1 Table — (DOCX) [file pntd.0009571.s002.docx]

**S1 Table.** Results of anti-*Toxocara* IgG antibodies titers and avidity index (AI) of IgY in 280 pregnant women (14 to 43 years old).

| Samples No. | Age | IgG Titers | log2 | Avidity index (AI) of IgY |
| --- | --- | --- | --- | --- |
| 1 | 20 | 400 | 8,6438 | 78,55 |
| 3 | 18 | 200 | 7,6438 | 64,84 |
| 52 | 18 | 1600 | 10,6438 | 83,87 |
| 64 | 19 | 3200 | 11,6438 | 68,17 |
| 65 | 19 | 3200 | 11,6438 | 48,51 |
| 66 | 18 | 3200 | 11,6438 | 77,37 |
| 69 | 32 | 400 | 8,6438 | 79,06 |
| 74 | 22 | 200 | 7,6438 | 83,48 |
| 81 | 18 | 400 | 8,6438 | 60,79 |
| 85 | 19 | 1600 | 10,6438 | 57,91 |
| 95 | 21 | 800 | 9,6438 | 75,64 |
| 105 | 33 | 200 | 7,6438 | 67,31 |
| 108 | 41 | 200 | 7,6438 | 82,23 |
| 121 | 23 | 400 | 8,6438 | 62,62 |
| 126 | 18 | 1600 | 10,6438 | 69,49 |
| 138 | 30 | 800 | 9,6438 | 72,48 |
| 150 | 25 | 200 | 7,6438 | 64,76 |
| 167 | 36 | 400 | 8,6438 | 74,61 |
| 174 | 25 | 200 | 7,6438 | 68,93 |
| 193 | 27 | 200 | 7,6438 | 79,86 |
| 197 | 26 | 1600 | 10,6438 | 84,92 |
| 200 | 37 | 400 | 8,6438 | 60,88 |
| 206 | 19 | 400 | 8,6438 | 73,2 |
| 212 | 24 | 400 | 8,6438 | 70,94 |
| 218 | 18 | 800 | 9,6438 | 75,16 |
| 232 | 28 | 200 | 7,6438 | 82,45 |
| 235 | 28 | 200 | 7,6438 | 69,5 |
| 236 | 18 | 400 | 8,6438 | 62,36 |
| 240 | 41 | 200 | 7,6438 | 63,93 |
| 256 | 25 | 200 | 7,6438 | 63,84 |
| 263 | 26 | 200 | 7,6438 | 81,8 |
| 266 | 25 | 200 | 7,6438 | 73,6 |
| 270 | 39 | 400 | 8,6438 | 75,09 |
| 279 | 27 | 200 | 7,6438 | 82,33 |
| 266 | 25 | 200 | 7,6438 | 45,9 |
| 270 | 39 | 400 | 8,6438 | 87,77 |
| 279 | 27 | 200 | 7,6438 | 56,71 |
